# Unlocking delocalization: how much coupling strength is required to overcome energy disorder in molecular polaritons?†

**DOI:** 10.1039/d4sc07053d

**Published:** 2025-02-03

**Authors:** Tianlin Liu, Guoxin Yin, Wei Xiong

**Affiliations:** a Department of Chemistry and Biochemistry, University of California San Diego La Jolla CA 92093 USA w2xiong@ucsd.edu; b Materials Science and Engineering Program, University of California San Diego La Jolla CA 92093 USA

## Abstract

Polaritons, quasiparticles formed from the collective strong coupling of light and matter, have been shown for their capability to modify chemical reactions, energy and charge transport – amazing features that can revolutionize the way we control molecular properties. Many of these features originate from the delocalization of polaritons, *i.e.*, polaritons possess delocalized wavefunctions, which is one of their hallmarks. Furthermore, polariton delocalization has long been assumed to be robust against disorder that is ubiquitous in chemical systems, without being fully checked. Herein, we examined the criteria to ensure delocalization in molecular polaritons, and this study reveals that transition energy disorder destroys delocalization of polaritons. In order to mitigate the impact of disorder and restore delocalization, the collective coupling strength needs to exceed four times the standard deviation of the energy disorder linewidth. This observation indicates a more stringent criterion for preserving the unique delocalization characteristics of polaritons compared to the conventionally adopted standard (Rabi splitting larger than photonic and molecular spectral linewidths). This work sheds light on previous polariton dynamic studies performed by our group and others, explaining why the onset of Rabi splitting capable of modifying dynamics is bigger than the strong coupling criteria, and it provides an important threshold to reach polariton delocalization for chemical and material research under strong coupling.

## Abstract 2

Polaritons lose delocalization in energetically disordered systems. A large Rabi splitting about 3–4 times of the inhomogeneous linewidths is required to restore delocalization. This study can guide future rational experiment designs.

## Graphical abstract

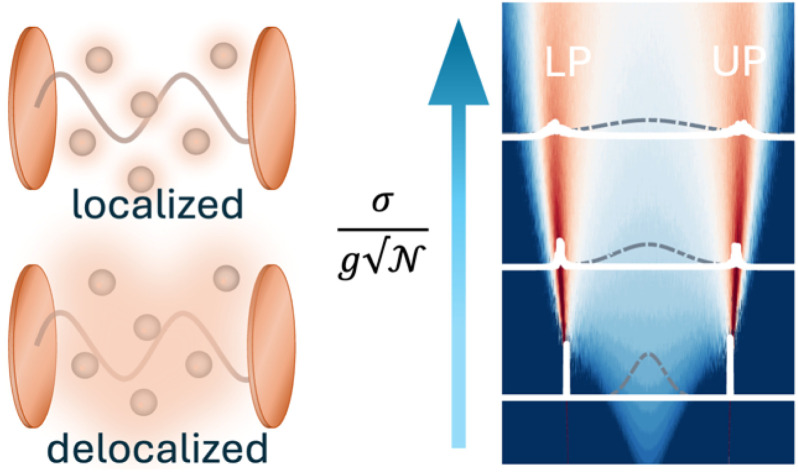

## Introduction

Polaritons,^1–5^ hybrid quasiparticles between photons and matter, have recently shown their potential in modifying chemical reactions and energy transfer.^4,6–14^ While the number of reports of polariton-induced modifications continues to grow, several irreproducible results and reinterpretations of results from non-polaritonic perspectives have led to skepticism and concerns.^15–17^ It is thereby important to reexamine the criteria of polariton formation and scrutinize their unique properties. Polaritons are formed under the so-called collective strong light-matter coupling conditions – when N quantum emitters, such as molecular transitions, and a cavity mode coherently exchange energy at a rate faster than their dissipation rates.^13^ It should be mentioned that strong coupling is typically claimed in experiments when the peak separation of polaritons (Rabi splitting, *Ω*) is larger than the linewidths (full-width-of-half-maximum, FWHM) of the molecular and cavity modes, since they are experimentally observable.^18^ Such a hybridization renders polaritons able to mix both light and matter properties *via* delocalized wavefunctions. Therefore, delocalization, that polariton wavefunctions are shared among many individual molecular wavefunctions, has been viewed as a key property leading to considerable enhancements of energy transmission,^9,10,12,19–21^ and subsequently influencing reactions. Recently, the investigation of the critical role of delocalization further extended to dark states.^22–24^

The delocalized nature of polaritons is concluded from an ideal system where all molecular modes emit at the same frequency (homogeneous limit).^25^ However, in many polaritonic systems under investigation, energy disorder (inhomogeneous limit) exists,^16,26–33^*i.e.* molecular transitions occur at different frequencies influenced by local environments. For example, the strong coupling of water stretching modes can be achieved (*Ω* = 500–800 cm^−1^) compared to its FWHM of *ca.* 400 cm^−1^ and has been reported to modify reactions or ion transport.^27,28,34,35^ However, inhomogeneous broadening significantly contributes to the total linewidth of water vibrational modes, which may deteriorate polariton characteristics, including delocalization.

Although it has been shown that energy disorder could influence polariton properties, including altering excitation lifetimes^36^ and accelerating decoherence,^37,38^ the premise that delocalization is robust against disorder has been widely assumed. This premise was supported by a seminal paper in 1995,^39^ in which Houdré *et al.* showed that the Rabi splitting (*Ω*) and linewidths (FWHM) of polariton states are generally immune to inhomogeneity, as long as the Rabi splitting is larger than the linewidths of the molecular absorption transition. The disorder only disrupts the symmetry of dark modes, resulting in slightly optically bright dark states.^11^ In a lossless cavity with an inhomogeneous distribution of transition frequencies, defined as 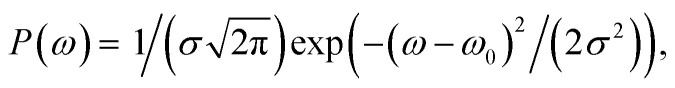 where *ω*_0_ and *σ* are the center and the standard deviation of the distribution, and 
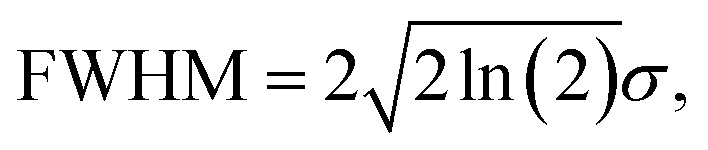
 this criterion can be translated to 
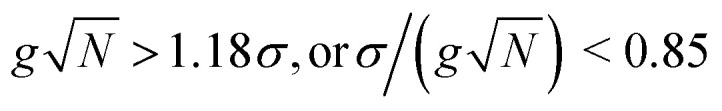
 (ref. 18) where *g* is the single molecule coupling strength and *N* is the number of molecules, which together describe *Ω* approximately as 
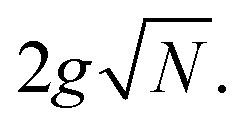
 This criterion 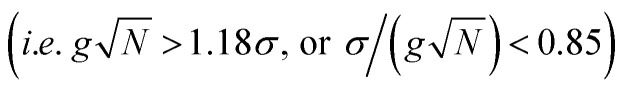 will be referenced frequently hereafter.

However, recent spectroscopic studies involving samples with high energy disorder reported that the transient signals of polaritons highly resemble those originating from the corresponding molecular absorption spectra filtered by polariton transmission spectra.^40–44^ These results hinted that under high disorder conditions, polaritons may behave similarly to localized molecules. Currently, a critical question – at the inhomogeneous limit, how delocalized are polaritons – remains largely unexplored in the context of chemistry. In this work, we investigated this question by solving the disordered Tavis–Cummings model,^45^ and found a critical threshold ratio 
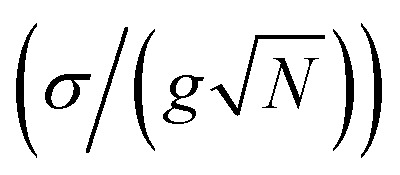
 of 0.25, below which delocalization is preserved in polaritons. Notably, this indicates that a Rabi splitting approximately three times larger than the widely adopted strong coupling criterion 
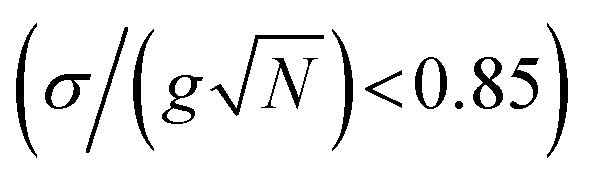
 is required to maintain delocalization in polaritonic systems with energy disorder.

## Results and discussion

We used the Tavis–Cummings model to describe the collective strong coupling in the single excitation space under the rotating wave approximation, and the Hamiltonian^46^ is shown in eqn (1):1
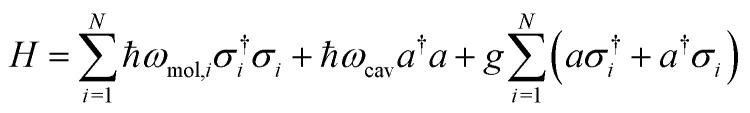


Specifically, the collective interaction is between an ensemble of *N* molecular transitions (|*φ*_mol,*i*_〉), indexed by *i* = 1, 2, …, *N* and described by two-level systems, and a single mode of a quantized cavity mode (|*φ*_cav_〉). The ground and excited states of the *N* molecules are separated by transition energies of *ω*_mol,*i*_ and connected by raising and lowering operators, *σ*^†^_*i*_ and *σ*_*i*_. The light field is quantized at *ω*_cav_ by photonic creation and annihilation operators, *a*^†^ and *a*. The distribution center of molecular transition frequencies and the cavity mode are on resonance if not otherwise stated, and the individual light–matter interaction strength is *g*.

By diagonalizing the Hamiltonian in eqn (1), *N* + 1 eigenvalues can be solved. The corresponding eigenstates (*ψ*^(*m*)^, *m* = 1, 2, …, *N* + 1) are represented by a linear combination of *N* molecular transitions (|*φ*_mol,*i*_〉) and the cavity mode (|*φ*_cav_〉), as shown in eqn (2):2
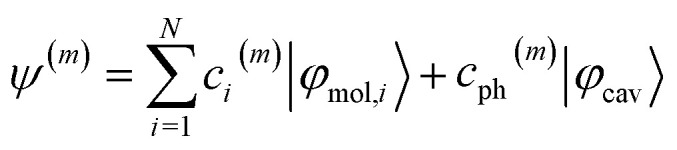


We then examined the weights of different components of polariton wavefunctions, which are so-called Hopfield coefficients 
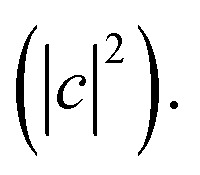
 Firstly, we calculated polariton spectra using the photonic weights. The eigenstates with the highest photonic weights above and below the cavity resonance energy are identified as upper (UP) and lower polaritons (LP) respectively. In contrast, the dark modes (DK) have minimal photonic weights. Secondly, we quantified the delocalization of polaritons among matter using the normalized inverse participation ratio (nIPR),^41,47^ as defined in eqn (3):3
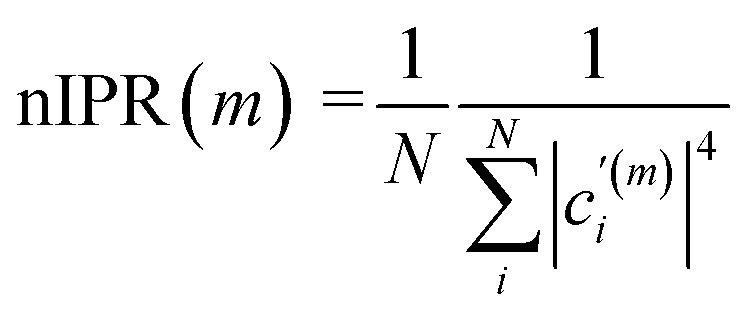


The 
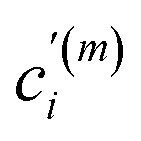
 is a modified linear combination coefficient of the *i*-th molecular transition in the *m*-th eigenstate, where the eigenvector of the *m*-th state is normalized to 1 after excluding its photonic entry. Furthermore, the nIPRs are normalized by the number of molecules, *N*, such that their values range between 1/*N* and 1, denoting complete localization and delocalization, respectively. More details can be found in Section S1 of the ESI.†

**Fig. 1:**
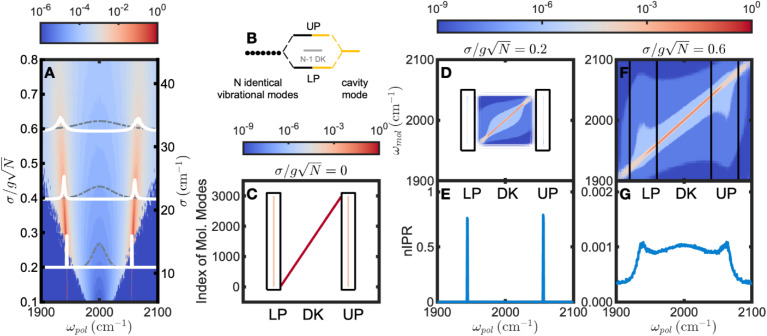
Evolution of polaritons as a function of energy disorder. (A) Spectra of light–matter coupled systems involving different disorders (*σ*). The spectral intensities are determined by the photonic weights, where blue corresponds to the weakest intensity and red is the strongest. The gray dashed lines represent inhomogeneous energy distributions of molecular transitions, and the white solid lines show the corresponding polariton spectra. As disorder increases, the polaritons retain their spectral signatures with broadened lineshapes. (B) and (C) schematically demonstrate an ideal case of strong coupling with no inhomogeneity. (B) shows that *N* identical molecular vibrational modes collectively interact with a resonant cavity mode, and (C) shows that each molecular mode (*y*-axis, indexed 1 to 3000 to distinguish the energetically identical molecules) equally contributes to the lower (LP) and upper polariton (UP) states, reflected by the vertical pink lines. Note that because each molecule is identical in frequency, they are differentiated by indices along the *y*-axis. Similarly, the energetically degenerate dark modes (DK) are spread along the *x*-axis, illustrating their one-to-one correspondence with nascent molecular modes. (D) and (F) demonstrate the contribution from each molecular transition (*y*-axis, binned by energy) to new eigenstates at 
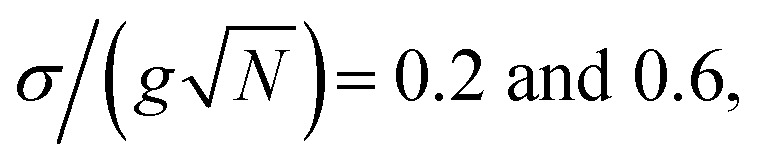
 respectively. As seen in (D), at 
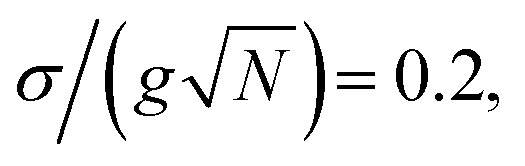
 both LP and UP are delocalized among molecules, indicated by the uniform blue-colored vertical lines along the *ω*_mol_ axis (highlighted by the black boxes), while DK are localized to the molecular transitions sharing similar frequencies, indicated by the red line along the diagonal. This is further confirmed by the nIPR values (E) that those of UP and LP are close to 1, and those of DK are essentially 0. By contrast in (F), at 
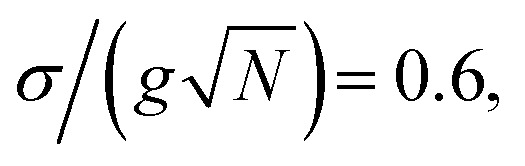
 both polaritons and dark modes are localized around the molecular transitions of close-by frequencies, represented by the diagonal line, and further confirmed by the nIPR values in (G), which are all nearly zero. The color bar indicates the magnitudes of molecular contributions of C, D and F on a logarithmic-scale from blue to red. Parameters: *ω*_mol,0_ = *ω*_cav_ = 2000 cm^−1^, *g* = 1 cm^−1^ and *N* = 3000.

We first validated the conclusion of Houdré’s work: in the strong coupling regime, polariton spectra remain qualitatively similar (Fig. 1A). A higher disorder (*σ*) leads to a slightly increased splitting and decreased optical brightness, corresponding to reduced photonic weights of polariton wavefunctions. In addition, linewidth broadening of polaritonic states is observed as a result of coupling between the cavity mode and detuned molecules, aligning well with previous theory and experiments.^38,46,48–50^

Despite the modest spectral evolution, the underlying composition of polaritons changes drastically with increasing disorder. To provide a comparison, we first show an ideal strong coupling case without disorder (Fig. 1B and C). Two bright polaritonic states emerge, evenly shifted from the resonance energy by 
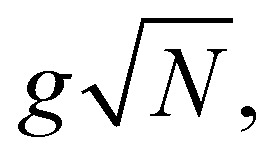
 whereas the energy levels of the remaining *N* − 1 dark modes remain unaltered. Fig. 1C illustrates the composition of the polaritonic wavefunctions from individual molecular wavefunctions. The matter component of polaritons involves all coupled molecular transitions uniformly (delocalized), as evident by the vertical pink lines at the polariton frequencies of *ω*_pol_ = 1944 and 2056 cm^−1^, respectively. In contrast, dark modes at 2000 cm^−1^ are degenerate, and the red line lying along the diagonal area indicates complete localization, with one-to-one correspondence with bare molecular transitions.

Subsequently, we varied the disorder (*σ*). With a small disorder of 
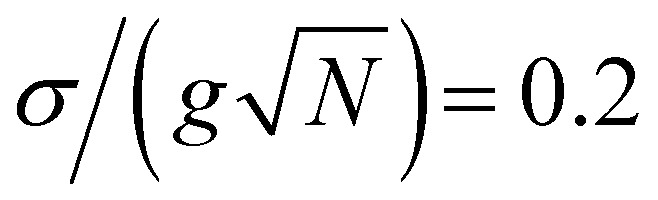
 (Fig. 1D), the distribution of polariton wavefunctions is still delocalized as manifested by uniform light-blue-colored vertical shadings (highlighted by black rectangular boxes), whereas similar to Fig. 1C, the dark modes are localized to limited molecules, indicated by the diagonal distribution that manifests as a red line. Additionally, the nIPRs of polaritons are calculated to be *ca.* 0.8 (Fig. 1E), agreeing with their delocalized nature, while the nIPRs of dark modes are negligible, characteristic of localized states. However, with a large disorder of 
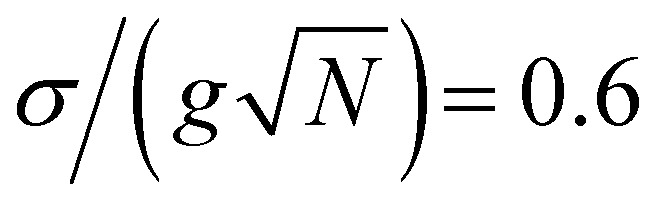
 (Fig. 1F), localized polaritons are revealed by their diagonal matter distribution, again appearing as a red diagonal line. Moreover, Fig. 1G shows nIPRs of *ca.* 0.001 through the spectrum. Both results signify that polaritons become analogous to dark modes, which are composed of molecular modes with similar energies. This observation is consistent with prior work that the idealness of polaritons is compromised by energy disorder.^51^ Noticeably, both scenarios (Fig. 1D–G) fall into the strong coupling regime according to the conventional standard 
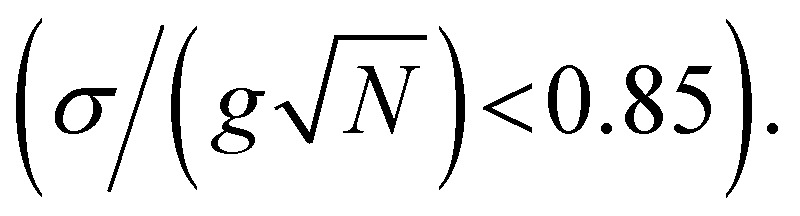
 Therefore, it is noteworthy that, even under strong coupling, polaritons may lose delocalization due to high energy disorder.

**Fig. 2:**
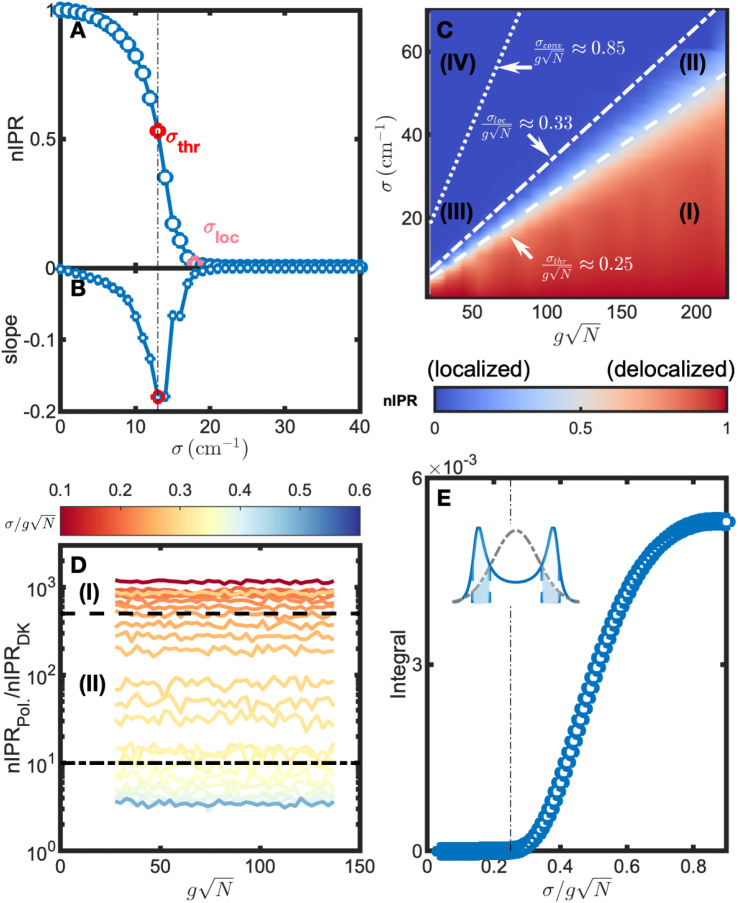
Delocalization threshold. (A) The nIPR of polaritons for coupled systems (*g* = 1 cm^−1^) with different disorders (*σ*). The transition threshold of *σ*_thr_ = 13 cm^−1^ is identified through the point (red in B) where the decline of nIPR is the fastest, and labeled in red. A second threshold, labeled in pink, is at *σ*_loc_ = 18 cm^−1^, above which the nIPR values are nearly zero. (C) shows the nIPR of polaritons as a function of both collective coupling strengths 
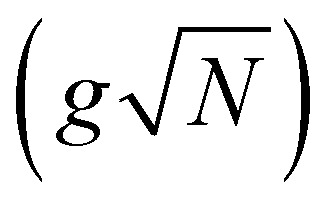
 and disorders (*σ*). The blue and red shadings correspond to localized and delocalized polariton wavefunctions, respectively. The dashed line between (I) and (II) indicates the delocalization threshold 
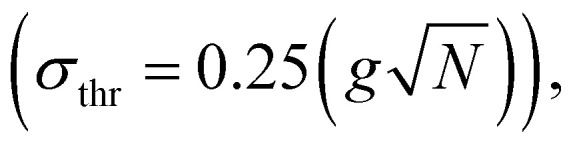
 the dot-dashed line between (II) and (III) indicates the second threshold 
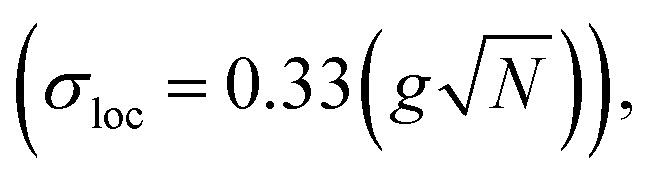
 and the dotted line between (III) and (IV) indicates the conventional strong coupling criterion 
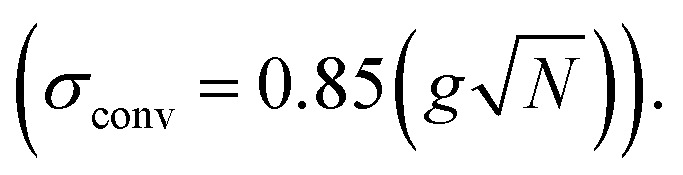
 Both (II) and (III) are in the conventional strong coupling regime; however, polaritons in the region (II) remain partially delocalized while polaritons in the region (III) become localized, analogous to dark modes. (D) Calculated nIPR ratios between polaritons (average of LP and UP) and dark modes under different 
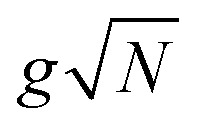
 and *σ* conditions. These nIPR ratios decrease as 
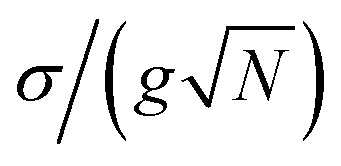
 increases. The dashed line indicates an nIPR ratio of 500 that corresponds to 
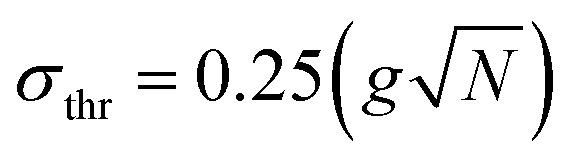
 in (C), signifying that polaritons in region (I) are delocalized. The dot-dashed line indicates an nIPR ratio of 10 that corresponds to 
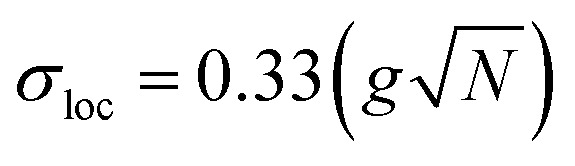
 in (C), above which polaritons maintain partial delocalization. The moderate noise arises from the random sampling of molecular transition energies in our simulations, and 100 repeated runs have been performed to ensure sufficient sampling and minimize noise. Parameters: *ω*_mol,0_ = *ω*_cav_ = 2000 cm^−1^ and *N* = 3000. (E) shows the localized molecular contribution. The inset of (E) provides a schematic illustration of the filter effect, where the blue solid line shows a polariton spectral window with blue dashed lines indicating its FWHM, and the gray dot-dashed line shows the molecular absorption spectrum. Points in (E) represent integrals of the filtered molecular absorption spectra within the light-blue shaded areas, which remain negligible approximately until 
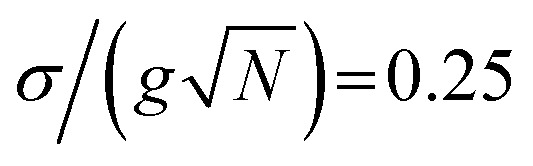
 (indicated by the black dot-dashed line), increase rapidly afterwards, and reach a plateau at around and beyond 
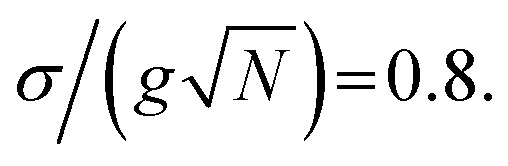

By exploring how nIPRs of polaritons change with energy disorder at various coupling strengths, we found a threshold when polaritons evolve from delocalized to localized. For example, Fig. 2A shows a representative nIPR curve as a function of *σ* with a collective coupling strength 
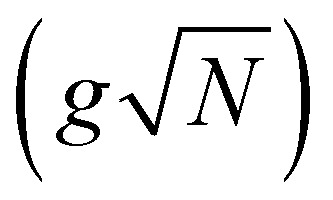
 of ∼55 cm^−1^. For *σ* below 10 cm^−1^, the nIPRs of polaritons gradually decrease from 1 to *ca.* 0.8 when *σ* increases. A subsequent rapid decline occurs when *σ* reaches *ca.* 13 cm^−1^, marking a sharp shift towards localization. Therefore, a sharp transition threshold of *σ*_thr_ = 13 cm^−1^ is identified, based on where the decline of the nIPR is the fastest (Fig. 2B). Beyond this, when *σ* is greater than 18 cm^−1^, the nIPRs remain close to 0, and thus polaritons become fully localized states. As a result, *σ* = 18 cm^−1^ can be regarded as a second threshold, *σ*_loc_. In the next paragraph, we show that *σ*_thr_ and *σ*_loc_ define the transitions where polaritons evolve from full to partial delocalization and subsequently to localization.

In fact, we found that these thresholds remain constant against 
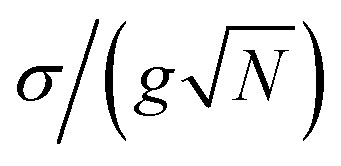
. As shown in Fig. 2C, the delocalization threshold (dashed line) is approximately at 
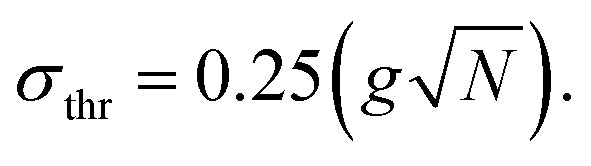
 Below this boundary, the nIPRs of polaritons (average of LP and UP) remain close to 1 (delocalized) regardless of coupling strengths or simulation sizes (Fig. S1†). Above this boundary (*σ* > *σ*_thr_), polaritons gradually lose their delocalization features. As shown in Fig. 2D, as energy disorder increases, the enhanced delocalization of polaritons (average nIPR of the LP and UP) decreases from 500 times to 10 times that of dark modes near the cavity resonance, signifying a shift towards localization. Full localization occurs at *ca.*
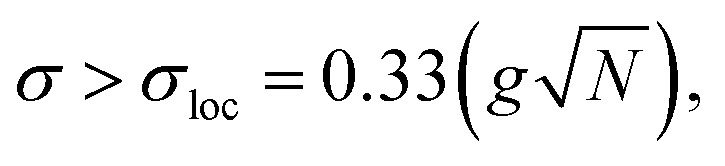
 beyond which (dot-dashed line in Fig. 2C and D) the polaritons' nIPRs are similar (<10 times enhancement) to those of dark modes. As a result, four regions are identified in Fig. 2C: (I) satisfies strong coupling and can guarantees the delocalization of polaritons. In the region (II), strong coupling is satisfied, and polaritons exhibit partial delocalization that contrast dark modes. However, polaritons in the region (III), although still in the strong coupling regime, become localized and similar to dark modes. The region (IV) is in the weak coupling regime. In summary, to achieve full delocalization in systems with energy disorder, the collective coupling strength 
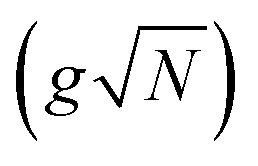
 needs to be four times the standard deviation (*σ*), while partial delocalization requires it to be three times the *σ*. These thresholds are more demanding than the classical criteria for strong coupling, which may be caused by the increase in localized oscillators at polariton transitions as explained in the next paragraph. In addition, these results are examined under detuned conditions (Section S3†), revealing the same delocalization threshold 
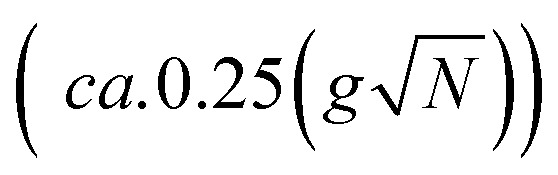
 for relatively large energy disorders (*σ* exceeding the detuning magnitude). However, the criterion can be relaxed for the polariton branch with greater photonic weight (*e.g.*, UP under positive detuning conditions), particularly when energy disorders are relatively small. This can be understood based on the fact that coherence among oscillators is mediated through the photonic mode.

To understand the origin of this delocalization threshold, we evaluated the contribution of localized oscillators by calculating the amount of molecular excitation through the polariton spectral window. We calculated the dot product of the normalized Gaussian-shaped molecular absorption spectrum and the analytical expression of the normalized polariton spectrum (reported in ref. 46 by Zeb, *cf.* eqn (25) and (30); see Section S1† for more details),^52^ and then integrated the filtered molecular absorption spectrum along the frequency axis, as illustrated by the inset of Fig. 2E. The integrals are shown as a function of disorder to coupling strength ratio, 
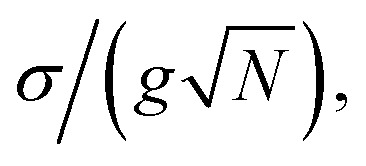
 in Fig. 2E. The contribution of localized oscillators is negligible when the relative disorder is small, *i.e.*
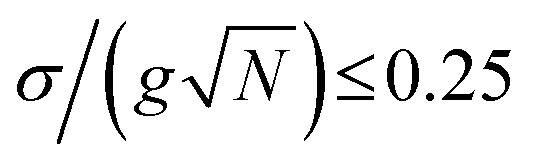
 (the boundary between region (I) and (II)) due to minimal spectral overlap. When 
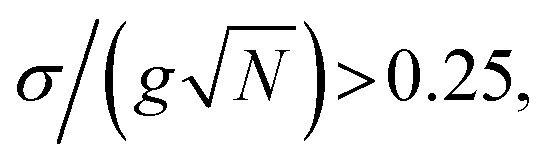
 the localized contribution rapidly increases and reaches a plateau at 
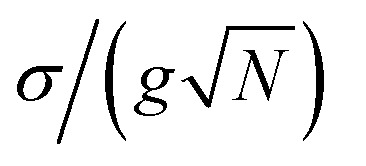
 beyond 0.8 (Fig. S2† shows the logarithmic-scale plot). To further understand this transition, we calculated the number of local transitions that can be excited through this polariton window. In this calculation, we use typical conditions where an ensemble of *N* ≈ 10^10^ molecular oscillators is required to achieve collective strong coupling.^13^ At 
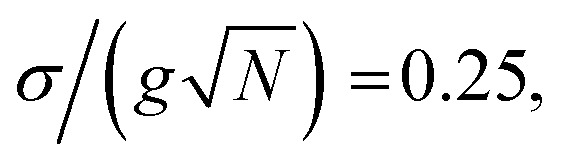
 a relatively large energy disorder, the probability of exciting localized transitions is 8 × 10^−6^, leading to 8 × 10^4^ locally excited molecules. In contrast, when the relative energy disorder is small, such as at 
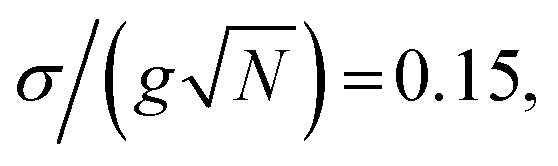
 the corresponding probability is 7 × 10^−12^, leading to less than 1 locally excited molecule. Thus, the smaller number of locally excited molecules necessitates that the polariton excitation is delocalized among matter wavefunctions with negligible localized excitations, which is in line with the delocalization pictured in the region (I) of Fig. 2C. Therefore, the origin of the new threshold is the drastic increase in local excitation through the polariton window.

Interestingly, we found that such a threshold 
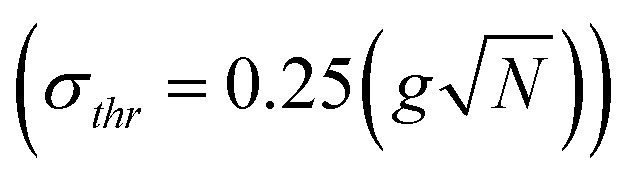
 also applies when considering the polariton dynamics. Here, we initialized a wavefunction (*ψ*(0)) in the photonic mode to mimic broadband coherent excitation of polaritons – a common scenario in ultrafast measurements.^1,10–13,42,43,53^ Then, we propagated it overtime using the time-dependent Schrödinger equation (*ψ*(*t*) = exp(−*iHt*/*ℏ*)*ψ*(0)), calculated the photonic and molecular populations 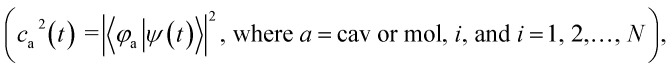 and evaluated the delocalization of the entire system using a similar nIPR for the system (see the ESI†)

**Fig. 3:**
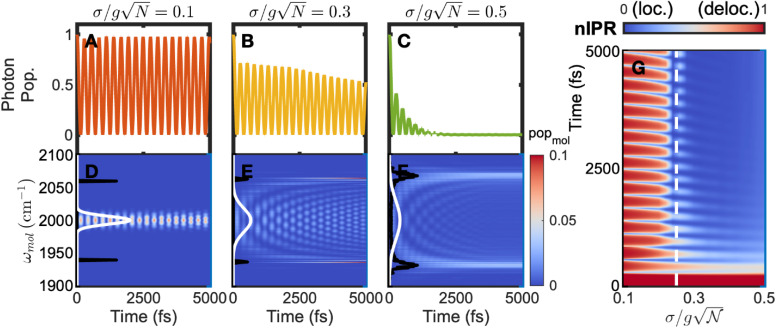
Effects of energy disorder on the temporal evolution of optical and molecular properties of polaritons. (A–C) The time-dependent population of the photonic state with 
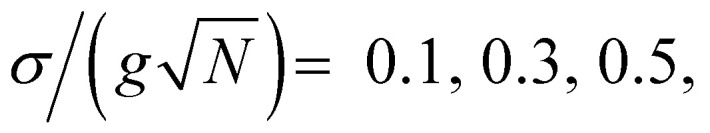
 respectively, showing that the photonic lifetime is significantly limited by increasing energy disorder. (D–F) The time-dependent population of excited molecular modes with 
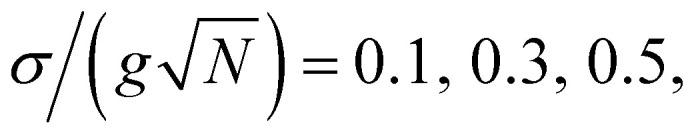
 respectively. The black lines illustrate the static polariton spectra, and the white lines show the inhomogeneous energy distributions of molecular absorption. Broadband excitation of all molecules is observed with small energy disorder in (D), whereas selected excitation of molecules with transition energies coinciding with polariton spectra is observed under large disorder conditions in (E and F) after a few oscillations. The excited molecules are binned by energy, and the blue to red shadings represents small to large excitation population. (G) The time evolution of the nIPR of the system calculated for different disorders 
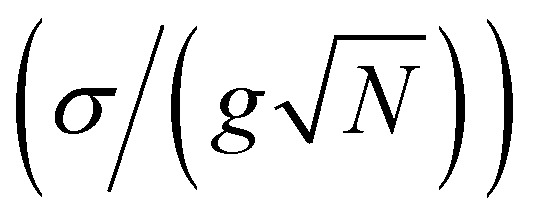
 with the blue and red shadings corresponding to localization and delocalization, respectively. The dashed line indicates 
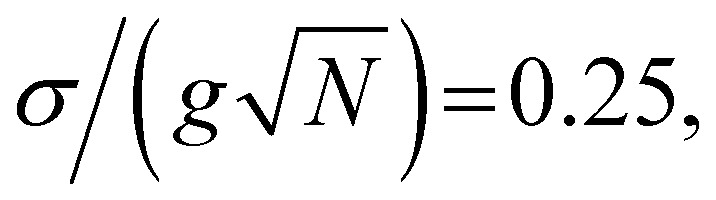
 above which delocalization is rapidly lost after two oscillations. Parameters: *g* = 0.6 cm^−1^ and *N* = 10 000.

The photonic population shows a smooth decrease in its lifetime as the disorder increases from 
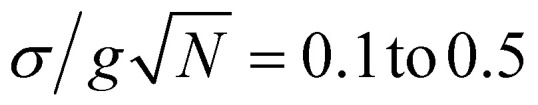
 (Fig. 3A–C). The limited polariton lifetime is a consequence of the loss of coherent return of energy from excited molecules to the photonic mode, when different oscillation frequencies destructively interfere.

Similarly, the delocalization loss exhibits a pattern akin to the photonic lifetime in response to energy disorder. As shown in Fig. 3D, at 
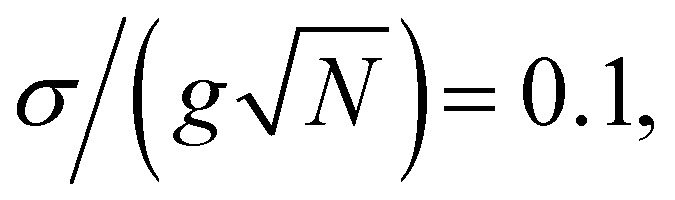
 when the energy periodically transfers back from the photonic mode to molecular modes, nearly all molecules are excited, and their distribution resembles the initial distribution of energy (white solid line). This scenario represents the strong-coupling phenomenon – the entire molecular ensemble is collectively and coherently populated when the system is excited. However, drastically different from 
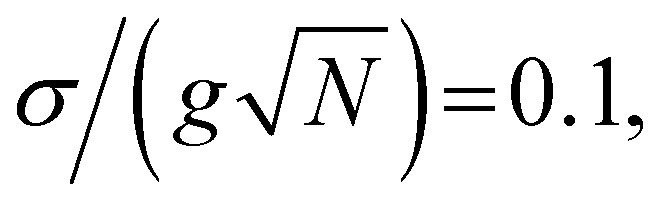
 as 
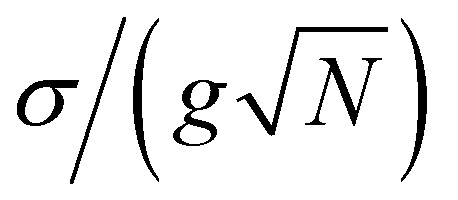
 increases to 0.3 and 0.5, such delocalization degrades quickly. In both cases, the initial broadband excitation quickly funnels the energy to molecular modes whose frequencies match those of polaritons, evident by the rapid redistribution of bright shadings (representing molecular excitation) in Fig. 3E and F from the center of the molecular absorption spectra (white solid lines) to the far split polariton regions (black solid lines) after a few initial oscillations. These excited molecules only account for a small fraction; however, their absorption frequencies coincide with the polariton transmission window, thus implying a filter effect by the polariton spectrum in line with prior work.^36,52,54,55^ We characterized the evolution of delocalization dynamics by surveying how the time-dependent nIPR evolves as a function of 
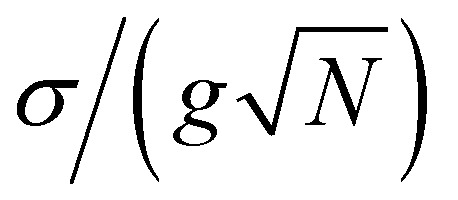
 (Fig. 3G). At low 
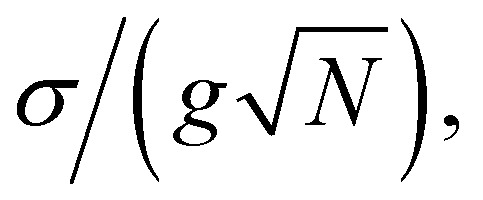
 nIPR oscillates and remains at a large value close to 1, indicating that the delocalization is preserved in the system. As the energy disorder increases, a sudden change in the nIPR dynamics occurs at 
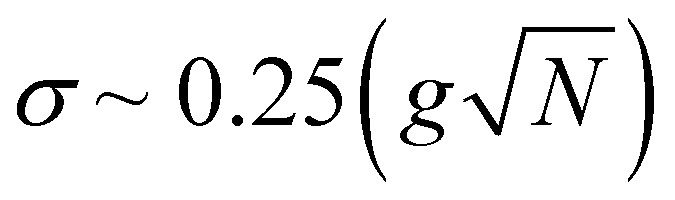
 (white dashed line), where the system loses its coherence at an early time. Therefore, 
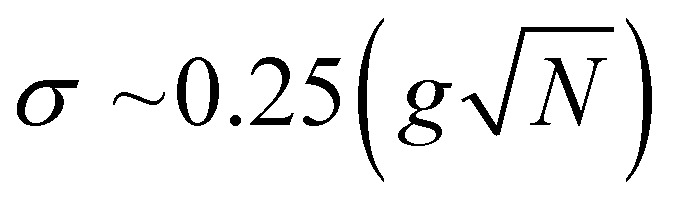
 can still serve as an empirical boundary for maintaining delocalization in the time domain. We note that the current dynamics simulation is undamped, *i.e.*, the lifetimes of cavity and molecular modes are set to infinity, for the purpose of understanding the role of delocalization. By incorporating the finite lifetimes of the molecular vibrational excited states and pure dephasing, we used a Lindblad master equation (Section S1.2 and 4 of the ESI†) and observed accelerated excitation relaxation and decoherence of the system as a result of additional dissipation channels.

## Conclusions

In summary, even if the Rabi splitting exceeds the linewidth of the molecular absorption spectrum, polaritons may not possess delocalized wavefunctions due to energy disorder. We found that the collective coupling strength 
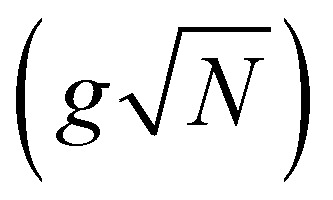
 needs to be 3 and 4 times the standard deviation (*σ*) of the energy distribution to obtain partial and full delocalization. This threshold remains valid for both static polariton wavefunctions, and the corresponding dynamics. Importantly, in many reported vibrational strong-coupled systems involving inhomogeneously broadened vibrational modes, *e.g.* water stretching modes,^27,28,34,35^ it is questionable whether delocalization is preserved. Relatedly, our group previously reported strong-coupling modified ultrafast molecular dynamics, *e.g.*, energy transfer, and we found that the coupling strength needs to be larger than the onset of strong coupling.^10,56^ This observation may be corroborated by that delocalization is required to modify molecular dynamics, yet satisfying the conventional strong coupling criterion alone may not ensure delocalization. The conclusion here may shed light on recent null effects, too.^15,57–60^ We note that in many inhomogeneous systems by reaching the new delocalization criteria, the systems are still within the strong coupling regime; however for systems with extreme inhomogeneous linewidths, such as liquid-phase water OH stretching modes, the systems may enter the ultrastrong coupling regime, which by itself warrants a detailed study in the future. In summary, for systems with inhomogeneous broadening, larger Rabi splittings are essential to secure delocalization, particularly when investigating the relationship between coupling strength and changes in chemical reactivity.

## Data availability

Simulation codes are available on request from the authors.

## Author contributions

T. L. conducted the simulation, data analysis and wrote the initial manuscript. G. Y. initialized the early simulation and participated in data analysis and manuscript writing. W. X. conceptualized the project, supervised the investigation and discussion, provided research funding and wrote the manuscript.

## Conflicts of interest

There are no conflicts do declare.

## Supplementary Material

SC-OLF-D4SC07053D-s001
