# Supplementary material for: Unlocking delocalization: how much coupling strength is required to overcome energy disorder in molecular polaritons?
Source: Chem Sci. 2025 Feb 3;16(11):4676–83. doi: 10.1039/d4sc07053d (PMC11817099; doi:10.1039/d4sc07053d)
Supplement: SC-016-D4SC07053D-s001 [file SC-016-D4SC07053D-s001.pdf]

Supplementary Information (SI) for Chemical Science.
This journal is © The Royal Society of Chemistry 2025

Unlocking Delocalization: How Much Coupling Strength can Overcome Energy Disorder in Molecular Polaritons?

Electronic Supplementary Information

Tianlin Liu,[1] Guoxin Yin,[2] and Wei Xiong[1,2*]

[1] Department of Chemistry and Biochemistry, University of California San Diego, La Jolla, CA 92093

[2] Materials Science and Engineering Program, University of California San Diego, La Jolla, CA 92093

Table of Contents

Section S1. Theoretical Method S2

Section S1.1. Static state simulation S2

Section S1.2. Dynamic simulation S3

Section S1.3. Simulation of the filter effect S4

Section S2. Figures S5

Figure S1. Simulation size dependence of disorder threshold ratio S5

Figure S2. Integral of molecular absorption filtered by the polariton spectrum S6

Section S3. Simulation size dependence, detuning, and dispersion S7

Figure S3. Detuning dependence of disorder threshold ratio S8

Figure S4. Dispersion curves under various energy disorder and detuning conditions................S10

Section S4. Population relaxation and pure dephasing S11

Figure S5. Comparison of relaxation and dephasing results inside and outside cavity................S11

Figure S6. Simulation size and coupling strength dependences of population relaxation S12

Figure S7. Simulation size and coupling strength dependences of dephasing S13

* Corresponding author email: w2xiong@ucsd.edu

Section S1. Theoretical Method

Section S1.1. Static state simulation

An ensemble of N molecules are indexed by i=1, 2, ..., N with vibrational ground and excited states $|0\rangle$ and $|1\rangle$ separated by transition energies of $\omega_{mol,i}$ and connected by raising and lowering operators $\sigma_i^\dagger$ and σ_i . The transition energies are inhomogeneously distributed around ω_0 following a Gaussian distribution $P(\omega_{mol,i})=1/(\sigma\sqrt{2\pi})\exp(-(\omega_{mol,i}-\omega_0)^2/(2\sigma^2))$, where the average and variance are ω_0 and σ^2 , respectively. The light field is quantized by the photonic creation and annihilation operators $a^\dagger$ and a with the energy ω_{cav}, and the light-matter interaction is g.

We restrict our simulation to the single excitation space, under the rotating wave approximation, in Equation (1), the Hamiltonian of the Tavis-Cummings model writes:

$$H = \hbar\omega_{cav}a^\dagger a + \sum_{i=1}^{N} \hbar\omega_{mol,i}\sigma_i^\dagger\sigma_i + g\sum_{i=1}^{N}(a\sigma_i^\dagger + a^\dagger\sigma_i)$$ Eq. (1)

By diagonalizing this Hamiltonian, N+1 eigenvalues and their corresponding eigenvectors can be solved. As shown in Equation (2), eigenvectors can be represented by a linear combination of the cavity mode ($|\varphi_{ph}\rangle$) and bare molecular transitions ($|\varphi_{mol,i}\rangle$)), and $|c|^2$ are usually called Hopfield coefficients.

$$|\psi\rangle = c_{ph}\,|\varphi_{ph}\rangle + \sum_{i=1}^{N} c_i\,|\varphi_{mol,i}\rangle$$ Eq. (2)

The spectrum of the light-matter coupled system can be calculated using $|c_{ph}|^2$, and the delocalized contribution from bare molecular transitions can be quantified using a normalized inverse participation ratio (nIPR) as defined in equation (3):

$$nIPR(m) = \frac{1}{\mathcal{N}}\frac{1}{\sum_i^{\mathcal{N}} |c_i'^{(m)}|^4}$$ Eq. (3)

The $c_i^{'(m)}$ is a modified linear combination coefficient representing contribution from the ith molecular transition to the mth hybridized state, where the eigenvector is renormalized to 1 after excluding its photonic entry (c_{ph}). Furthermore, the nIPR is normalized by the molecular ensemble size of N, such that its value ranges between 1/N (approaching 0 for large N) and 1, denoting completely localized and delocalized wavefunctions, respectively. Results presented in the main text are simulated employing N= 3000, and 100 repetitive simulations have been run independently and averaged to ensure sufficient sampling. For other ensemble sizes used in our static state simulations, a repeat number of 300000/N was chosen unless otherwise stated.

Section S1.2. Dynamic simulation

First, we initialize a wavefunction on the photonic mode, mimicking broadband excitation of polariton spectrum, and propagate it overtime using the time-dependent Schrödinger equation. The projection of the time-dependent wavefunction onto each bare molecular mode ($c_i(t)=\langle \varphi_{mol,i} | \varphi(t) \rangle$) is renormalized to unity and used to calculate the nIPR according to Eq. (4):

$$nIPR = \frac{1}{\mathcal{N}} \frac{1}{\sum_i^{\mathcal{N}} |c_i'(t)|^4}$$ Eq. (4)

Similar to the nIPR(m) defined in Eq. (3), the nIPR describing the system ranges between 0 and 1, representing localization and delocalization, respectively.

Next, we write the Lindblad master equation of this system in Eq. (5) for the population relaxation and decoherence simulation:

$$\dot{\rho} = -\frac{i}{\hbar}[H, \rho] + \sum_k \gamma_k (L_k \rho L_k^\dagger - \frac{1}{2}(L_k^\dagger L_k \rho + \rho L_k^\dagger L_k))$$ Eq. (5)

Here, ρ is the density matrix of the system, H is the Hamiltonian, and L_k are Lindblad operators representing some decoherence or relaxation processes that occur at rates of γ_k. Specifically, the L_k describing a pure-dephasing process for the kth molecule has the corresponding diagonal element of −1, while the remaining diagonal elements are 1 and off-diagonal elements are 0. The L_k describing a vibrational relaxation process from the kth molecular excited state to the ground state has the corresponding off-diagonal element of 1, while the remaining elements are 0. We also initialize a wavefunction in the coherent state of $|\varphi(0)\rangle = \frac{1}{\sqrt{N}} \sum_i^N |g_1, g_2, \ldots, e_i, \ldots, g_N\rangle$ to simulate the outside-cavity scenario for comparison. The inside and outside cavity comparison will be presented in Section S4 and Figure S7–9.

Section S1.3. Simulation of the filter effect

The analytical expression of the polariton spectrum ($A(\omega)$) is derived in Ref. 1 and given in Eq. (6):

$$A(\omega) = \frac{\omega_R^2 P(\omega)}{[\omega - \omega_c - F(\omega)]^2 + \pi^2 \omega_R^4 P(\omega)^2} \quad \text{Eq. (6a)}$$

$$F(\omega) = \pi \omega_R^2 P(\omega) erfi(\frac{\omega}{\sqrt{2}\sigma}) \quad \text{Eq. (6b)}$$

$$erfi(\omega) \equiv erf(i\omega)/i \quad \text{Eq. (6c)}$$

where ω_R is the collective coupling strength (approximated as $g\sqrt{N}$), $P(\omega)$ is the normalized molecular absorption spectrum of a Gaussian lineshape, $P(\omega)=1/(\sigma\sqrt{2\pi})\exp(-(\omega-\omega_0)^2/(2\sigma^2))$, where ω_0 is the center of molecular transition frequencies and ω_c is the cavity frequency (under resonant conditions, we set $\omega_0 = \omega_c = 0$ cm^{-1}), and the erfi function is the imaginary error function.

The dot product of the molecular absorption spectrum and the numerically normalized polariton spectrum is integrated along the frequency axis within the full-width-of-half-maximum (fwhm) of polariton bands (UP and LP). Specifically, the collective coupling is chosen as 50 cm^{-1}, thus the standard deviation of the Gaussian distribution changes according to the value of $\sigma/(g\sqrt{N})$, which is scanned between 0.04 and 0.9 with a step size of 0.01. In frequency axis spans from -100 to 100 cm^{-1}, with a frequency step of $0.01 \times \sigma/(g\sqrt{N})$ to guarantee sufficient number of points under the narrow polariton peaks for integration. The resultant integrals are plotted as a function of $\sigma/(g\sqrt{N})$ in Figure 2E in the main text. The localized contribution is negligible when $\sigma/(g\sqrt{N}) \leq 0.25$ due to minimal spectral overlap, above which the localized contribution rapidly increases (logrithmic plot in Figure S2).

Section S2. Figures (mentioned in main manuscript)

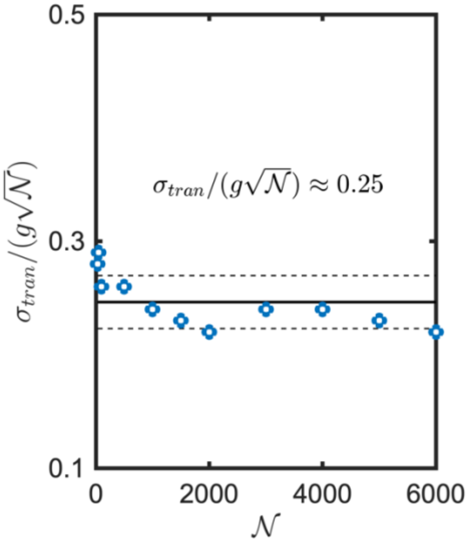

Figure S1. The threshold ratios for nIPR transitioning from delocalization to localization in terms of $\sigma/(g\sqrt{N})$ as a function of simulation size. All simulation results are averaged from N_{rep} trials such that $N_{rep} \times N = 300000$. Other simulation parameters are $\omega_{cav} = \omega_{mol,0} = 2000$ cm^{-1}, g = 1 cm^{-1}.

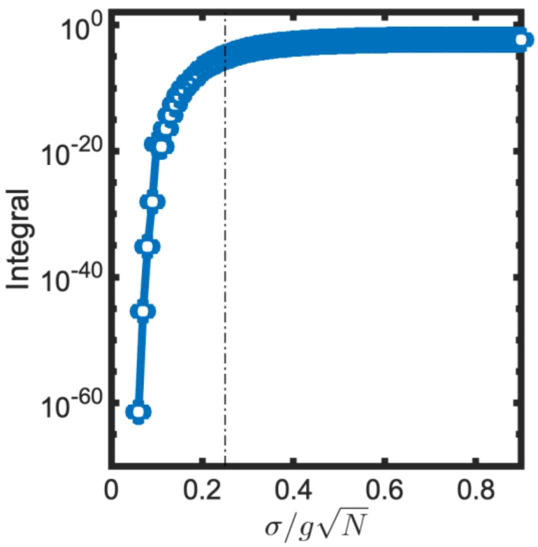

Figure S2. The logarithmic-plot of integrated localized molecular contribution by filtering the molecular absorption by a polariton spectral window. A linear-scale plot is shown as Figure 2E in the main text.

Section S3. Simulation size dependence, detuning, and dispersion

Several considerations should be addressed regarding the generalizability of our conclusion. The first concern is the robustness of the delocalization threshold ratio towards simulation size, which we explored by examining its ensemble size dependence. Shown in Figure S1, the threshold ratio of $\sigma/(g\sqrt{N})$ is ca. 0.3 when N= 30, which gradually approaches ca. 0.23 when ensemble sizes are larger than 1000. On average, the threshold is estimated to be ca. 0.25. Based on this, we predict that a similar transition of the delocalization nature would occur for a more realistic and larger system (N on the order of 10^6 and larger), when the ratio between the inhomogeneous linewidth and the collective coupling is between 0.2 and 0.3. In other words, the delocalization character of polaritons may be reached only when the separation between polariton peaks is 3–5 times larger than the linewidths of inhomogeneous broadening of molecular transitions.

The second question is whether the threshold ratio remains valid when the cavity frequency is detuned from the molecular modes. We found the threshold ratio depends on the extend of the detuning. As shown in Figure S3 with a representative detuning of 10 cm^{-1}, when the cavity detuning falls within the linewidth of the inhomogeneous broadening, the delocalization threshold ratio appears to be unaffected. In contrast, detuning starts to influence the delocalization threshold ratio once its magnitude exceeds the inhomogeneous broadening. Given that $\omega_{cav} > \omega_{mol,0}$, the delocalization property of UP has a greater tolerance for disorder, in contrast, the delocalization of LP is lost at an even lower $\sigma/(g\sqrt{N})$ ratio. This can be rationalized by the fact that UP is predominantly composed of photon when highly blue-detuned, allowing it to couple to all molecular modes and resulting in a higher level of delocalization in its molecular component. In contrast, LP is nearly matter-like, thus requiring a stronger coupling strength to reinforce delocalization. The detuning dependence suggests a possibility of enhancing the delocalization of one branch of the polaritons by tuning the cavity mode towards that specific direction. However, one should be cautious that detuning will eventually lead to decoupling between photon and matter, resulting in the disappearance of polaritons and pure excitation of either photon or matter.

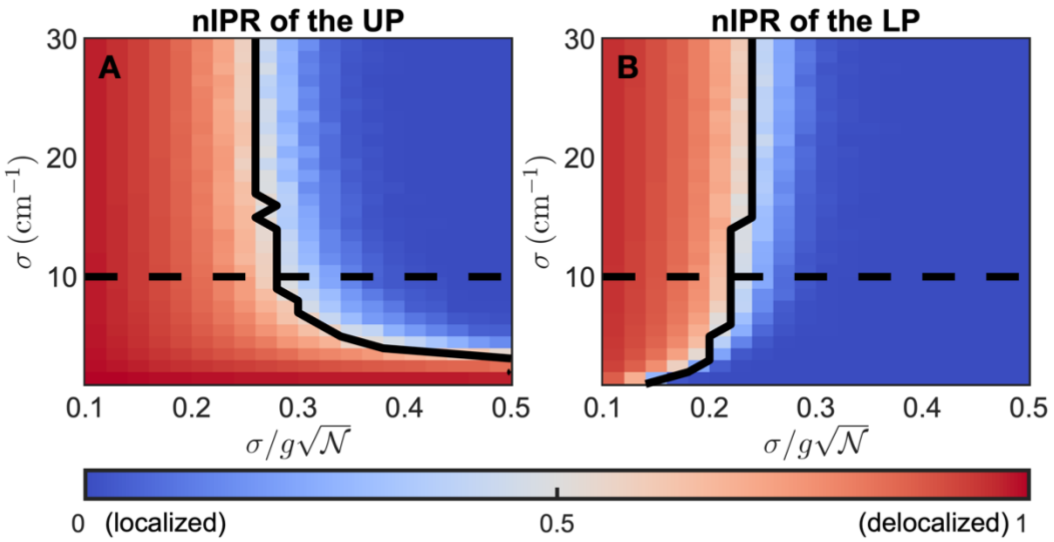

Figure S3. The variation of the normalized Inverse Participation Ratio (nIPR) of the upper polariton (UP, A) and lower polariton (LP, B) as a function of the inhomogeneous linewidth (σ) and its ratio with the collective coupling strength ($\sigma/(g\sqrt{N})$). The nIPR ranges from 1/N to 1, representing the fully localized and delocalized polariton wavefunctions. The black dashed lines indicate the magnitude of cavity detuning. The black solid lines indicate the delocalization threshold ratios under each disorder. Parameters used in this simulation include N= 3000, g = 1 cm^{-1}, ω_{cav} = 2010 cm^{-1}, and $\omega_{mol,0}$ = 2000 cm^{-1}, and the results are averaged from 100 repeats.

The third question deals with dispersion, which is elaborated in the main text, and here we provide more simulation results calculated with different $\sigma/(g\sqrt{N})$ ratios (Figure S4). We observed a positive correlation between the photonic weight of polaritons and their delocalization when investigating dispersion properties of polariton spectra and nIPRs under various detuning conditions. Four representative energy disorders are selected: $\sigma/(g\sqrt{N})$= 0.1 and 0.2 from the region (I), $\sigma/(g\sqrt{N})$ = 0.3 from the region (II), and $\sigma/(g\sqrt{N})$ = 0.4 from the region (III). In the region (I), polaritons show significant robustness of delocalization upon detuning and along the dispersion curve (Figure S4A and B). In contrast, the latter two (Figure S4C and D) exhibit weak polariton intensities and minimal nIPR at normal incidence. Larger incident angles yield a more photonic UP and result in higher delocalization (bigger nIPR). In addition, red-detuning (Figure S4, panel 3 of each condition) allows the LP to shift away from the dispersionless dark modes towards lower frequencies, recovering its photonic weight and delocalization, whereas the UP merges into the dark modes, yielding weaker spectral intensity and localized nIPRs.

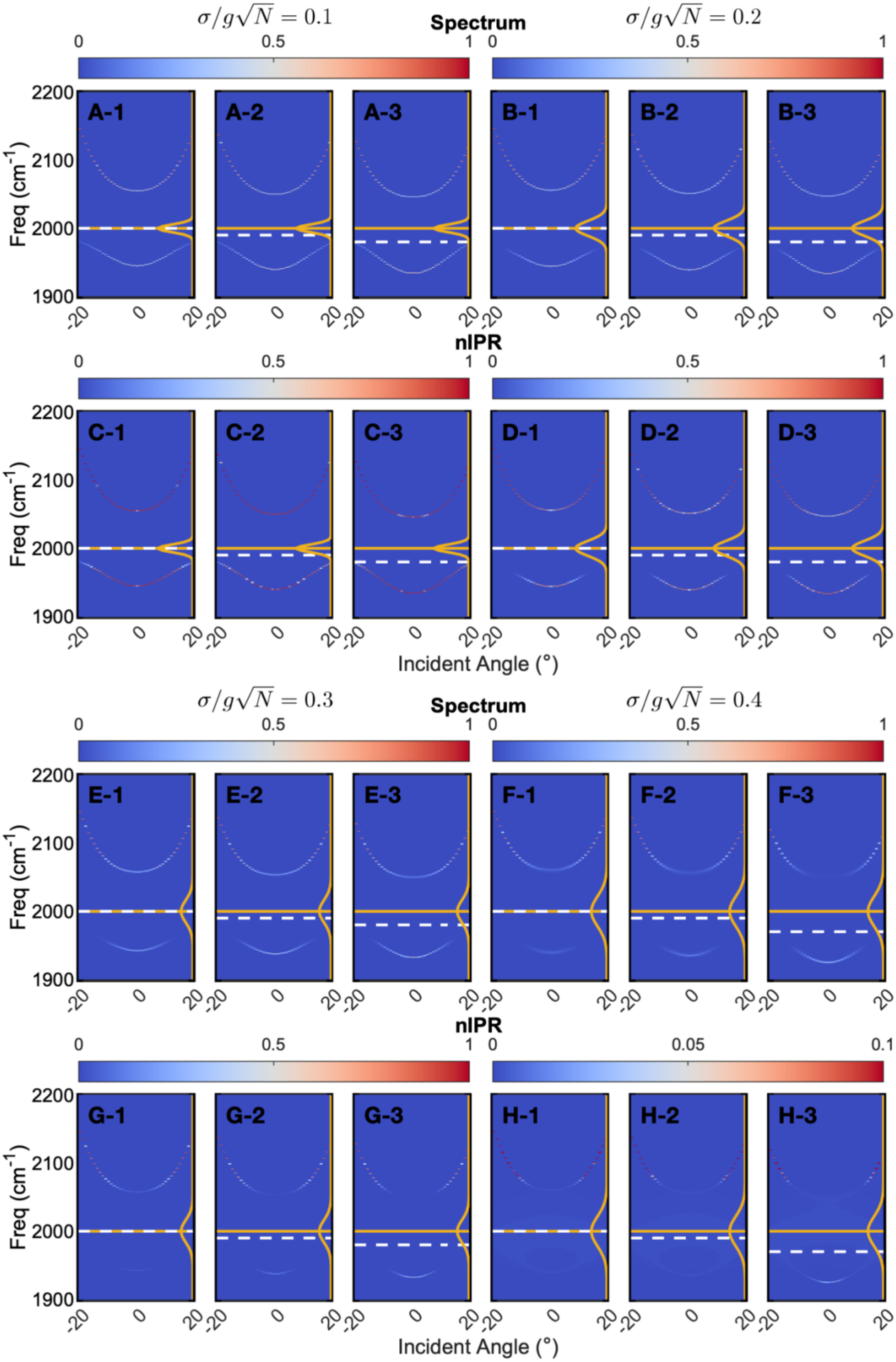

$\sigma/g\sqrt{N} = 0.1$
Spectrum
$\sigma/g\sqrt{N} = 0.2$
A-1
A-2
A-3
B-1
B-2
B-3
nIPR
C-1
C-2
C-3
D-1
D-2
D-3
Freq (cm^{-1})
Incident Angle (°)
$\sigma/g\sqrt{N} = 0.3$
Spectrum
$\sigma/g\sqrt{N} = 0.4$
E-1
E-2
E-3
F-1
F-2
F-3
nIPR
G-1
G-2
G-3
H-1
H-2
H-3
Freq (cm^{-1})
Incident Angle (°)

Figure S4. Dispersion curves of polariton spectra and delocalization as represented by nIPR calculated under various disorder conditions: $\sigma/(g\sqrt{N})$ = 0.1 (A, C), 0.2 (B, D), 0.3 (E,G), and 0.4 (F, H), respectively. Parameters: $\omega_{mol,0}$ = 2000 cm^{-1}, g = 1 cm^{-1} and N = 3000. White dashed lines represent cavity mode frequencies, and yellow solid lines represent molecular energy distributions and center frequencies.

Section S4. Population relaxation and pure dephasing

We found that the excited state lifetime of the coupled system can be lengthened by the lossless cavity compared to pure molecules, however, such an effect quickly disappears due to energy disorder, especially when $\sigma/(g\sqrt{N})$ exceeds 0.25. To simulate the relaxation dynamics, we assumed a vibrational excited state lifetime of ca. 5.3 ps, equivalent to a Lorentzian linewidth of 2 cm^{-1} due to lifetime broadening. An additional ground state entry is introduced, which becomes populated upon relaxation of vibrationally excited molecules. Figure S5A illustrates that the replenishment of the ground state is slowed down by the cavity via light-matter coupling, leading to a maximum of ca. 50% decrease in the exponential rate constant in the absence of energy disorder. Although it would be difficult to fabricate a perfect cavity in reality, it is possible to lengthen the excited vibrational lifetime if a high-quality factor cavity is utilized.

We also observed a similar trend in the decoherence dynamics. We employed a pure dephasing time of ca. 5.3 ps in the simulations, after which the density matrix transforms into a decoupled mixed state, alternatively speaking, polaritons decay into dark reservoir modes. The loss of coherence is evident by the reduced purity of the density matrix ($\mathrm{Tr}(\rho^2)$). Similarly, figure S5B shows that the decoherence is slowed down inside the cavity, with a maximum of ca. 40% decrease of the rate constant in the absence of energy disorder, whereas increasing disorder again attenuates the effect, gradually converging to the outside-cavity result.

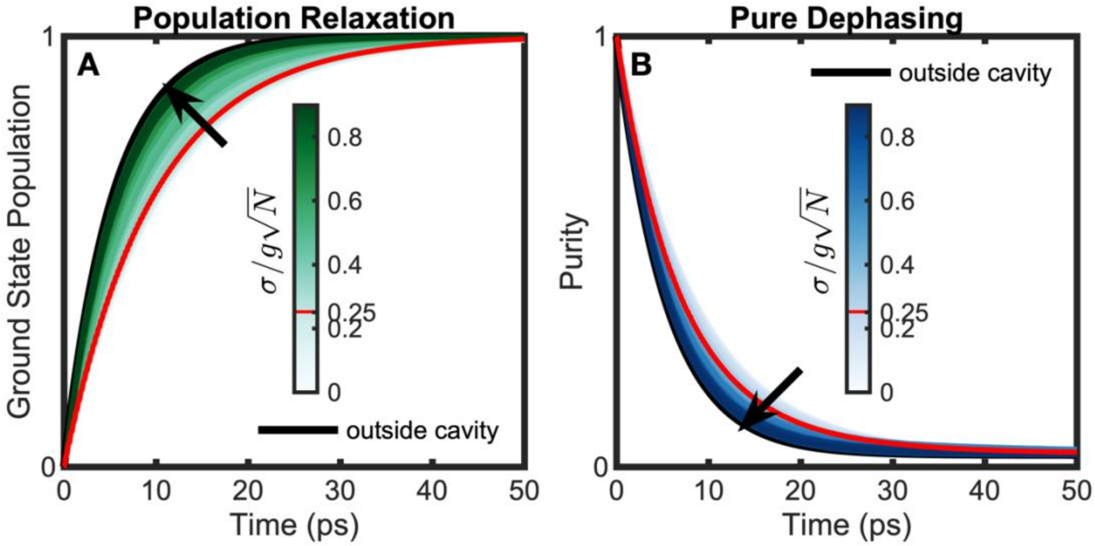

Figure S5. (A): Temporal profiles of ground state population calculated with increasing degrees of energy disorder (light to dark green) using a molecular vibrational excited state life of ca. 5.3 ps. (B) The purity of the time-dependent density matrixes calculated with increasing degrees of energy disorder (light to dark blue) using a pure dephasing time of ca. 5.3 ps. The black lines indicate the outside cavity results, and the red lines highlight the results of $\sigma/(g\sqrt{N})$ = 0.25. Parameters: g = 10 and N = 36.

It is also worth noting that we employed a small ensemble size of N= 36 to reduce the computational cost of dynamic simulations while using the Lindblad equation, and the convergence has been achieved. Shown in Figure S6, when keeping constant values of the Rabi splitting, energy disorder, and vibrational relaxation rate (or dephasing rate in Figure S7), different simulation sizes result in identical kinetic traces of the ground state population (or purity temporal profile in Figure S7). In addition, the overall dynamical trends remain unchanged when varying Rabi splitting as long as the $\sigma/(g\sqrt{N})$ is constant, although smaller Rabi splittings can lead to more pronounced oscillatory patterns.

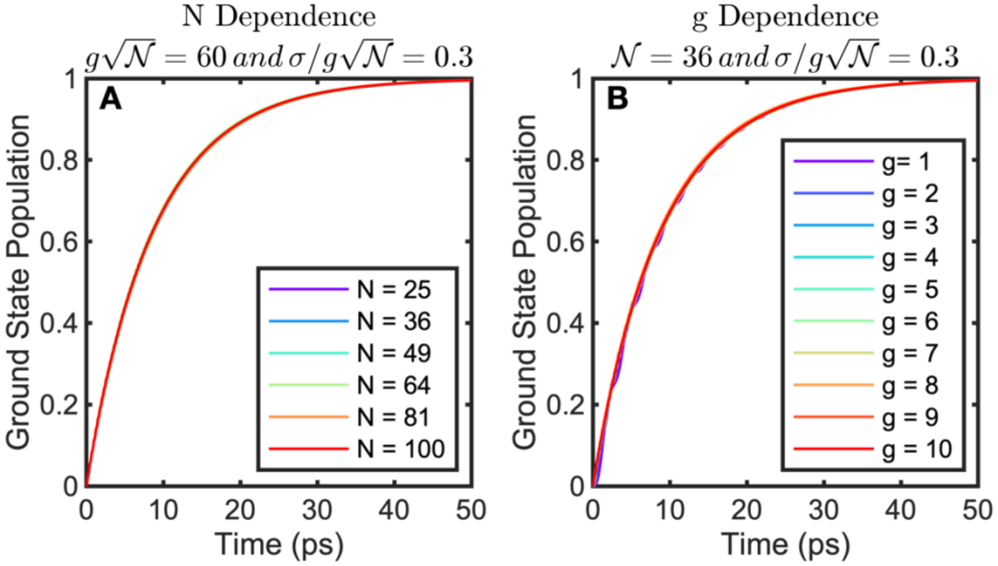

Figure S6. Temporal profiles of the ground state population calculated with (A): fixed collective coupling strength and disorder ratio, but varying ensemble size. (B): fixed ensemble size and disorder ratio, and varying coupling strength.

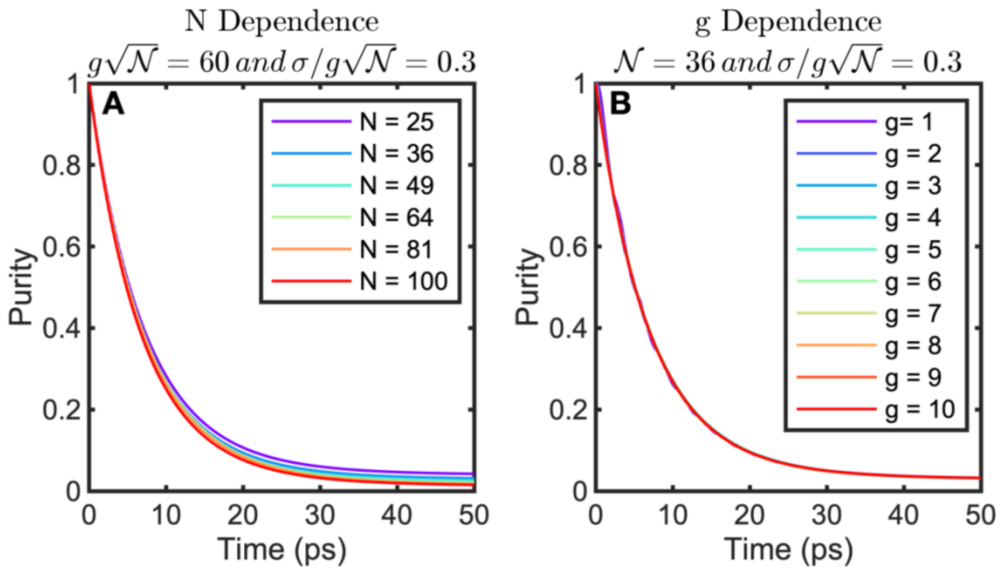

Figure S7. The purity of the time-dependent density matrix calculated with (A): fixed collective coupling strength and disorder ratio, but varying ensemble size. (B): fixed ensemble size and disorder ratio, and varying coupling strength.

References

1. M. A. Zeb, Analytical solution of the disordered Tavis-Cummings model and its Fano resonances, *Phys. Rev. A*, 2022, **106**, 063720.
